# 2,5-Di­meth­oxy­benzo­nitrile

**DOI:** 10.1107/S1600536813031309

**Published:** 2013-11-23

**Authors:** Bernhard Bugenhagen, Yosef Al Jasem, Thies Thiemann

**Affiliations:** a Fachbereich Chemie, University of Hamburg, Martin-Luther-King-Platz 6, 20146 Hamburg, Germany; b Department of Chemical Engineering, United Arab Emirates University, AL Ain, Abu Dhabi, United Arab Emirates; c Department of Chemistry, United Arab Emirates University, AL Ain, Abu Dhabi, United Arab Emirates

## Abstract

In the title mol­ecule, C_9_H_9_NO_2_, the non-H atoms are essentially coplanar with a maximum deviation of 0.027 (2) Å for the C atom of one of the methyl groups. In the crystal, the mol­ecules are arranged into centrosymmetric pairs *via* pairs of C—H⋯O and C—H⋯N inter­actions whereas π–π stacking inter­actions between the benzene rings [centroid–centroid distance 3.91001 (15) Å] organize them into polymeric strands propagating along the *a-*axis direction. There is a step of 0.644 (2) Å between the two planar parts of the centrosymmetric pair. In neighboring strands related by the *n*-glide operation, the aromatic rings are tilted by 29.08 (2)°.

## Related literature

For the use of the title compound as a key reagent in the synthesis of pharmaceutically active heterocycles, see: Bergeron *et al.* (2006 ▶); Delgado *et al.* (1987 ▶). For another method of preparation of the title compound, see: Ushijima *et al.* (2012 ▶). For the crystal structures of aromatic nitriles, see: Buschmann *et al.* (1995 ▶); Zabinski *et al.* (2007 ▶); Zanotti *et al.* (1980 ▶).
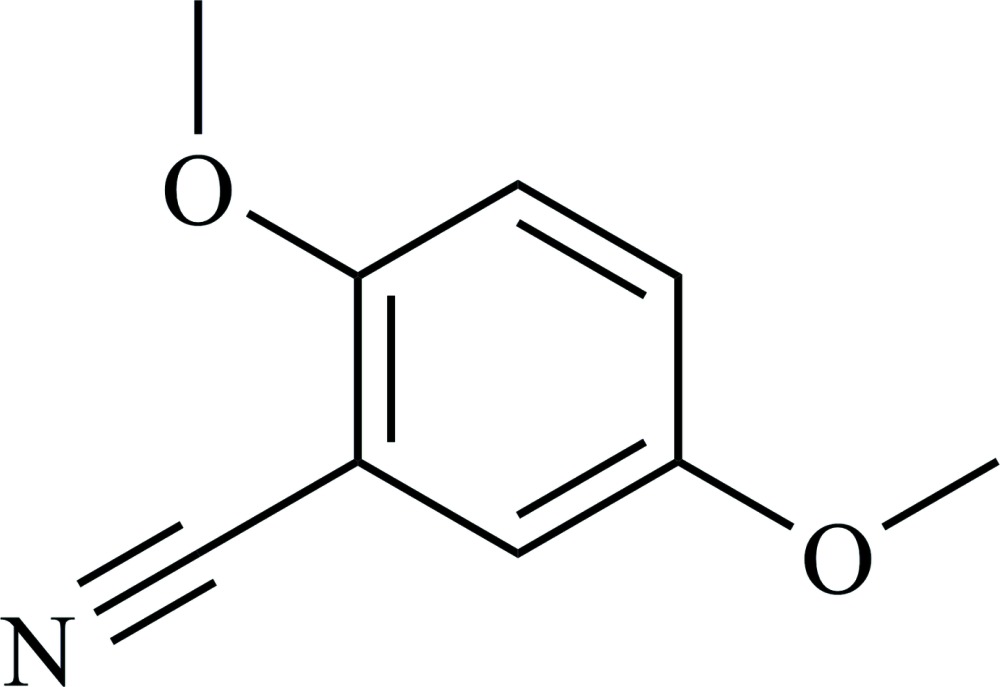


## Experimental

### 

#### Crystal data

C_9_H_9_NO_2_
*M* *_r_* = 163.17
Monoclinic, 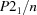

*a* = 3.91001 (15) Å
*b* = 11.3347 (4) Å
*c* = 17.8432 (6) Å
β = 93.400 (3)°
*V* = 789.40 (5) Å^3^
*Z* = 4
Mo *K*α radiation
μ = 0.10 mm^−1^
*T* = 100 K
0.60 × 0.25 × 0.23 mm

#### Data collection

Agilent SuperNova (Dual, Cu at zero, Atlas) diffractometer
Absorption correction: multi-scan (*CrysAlis PRO*; Agilent, 2013 ▶) *T*_min_ = 0.899, *T*_max_ = 1.000
3225 measured reflections
1785 independent reflections
1374 reflections with *I* > 2σ(*I*)
*R*_int_ = 0.026

#### Refinement

*R*[*F* ^2^ > 2σ(*F* ^2^)] = 0.044
*wR*(*F* ^2^) = 0.116
*S* = 1.06
1785 reflections
111 parameters
H-atom parameters constrained
Δρ_max_ = 0.21 e Å^−3^
Δρ_min_ = −0.24 e Å^−3^

### 

Data collection: *CrysAlis PRO* (Agilent, 2013 ▶); cell refinement: *CrysAlis PRO*; data reduction: *CrysAlis PRO*; program(s) used to solve structure: *SHELXS97* (Sheldrick, 2008 ▶); program(s) used to refine structure: *SHELXL97* (Sheldrick, 2008 ▶) within *OLEX2* (Dolomanov *et al.*, 2009 ▶); molecular graphics: *PLATON* (Spek, 2009 ▶); *Mercury* (Macrae *et al.*, 2008 ▶); software used to prepare material for publication: *SHELXL97* and *PLATON*.

## Supplementary Material

Crystal structure: contains datablock(s) I. DOI: 10.1107/S1600536813031309/gk2594sup1.cif

Structure factors: contains datablock(s) I. DOI: 10.1107/S1600536813031309/gk2594Isup2.hkl

Supplementary material file. DOI: 10.1107/S1600536813031309/gk2594Isup3.cml

Additional supplementary materials:  crystallographic information; 3D view; checkCIF report

## Figures and Tables

**Table 1:** Hydrogen-bond geometry (Å, °)

*D*—H⋯*A*	*D*—H	H⋯*A*	*D*⋯*A*	*D*—H⋯*A*
C8—H8*B*⋯O1^i^	0.98	2.65	3.428 (2)	136
C8—H8*B*⋯N1^i^	0.98	2.73	3.504 (2)	136
C9—H9*B*⋯N1^ii^	0.98	2.71	3.640 (2)	158

Symmetry codes: (i) 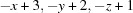; (ii) 
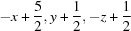
.

**Fig. 1.**
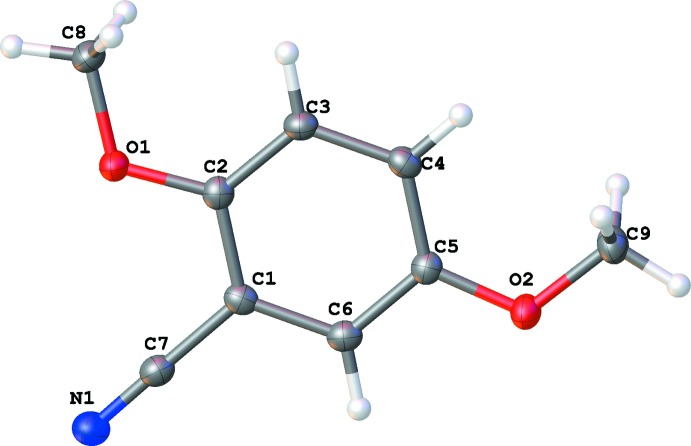
A view of the title molecule with displacement ellipsoids shown at the 50% probability level.

**Fig. 2.**
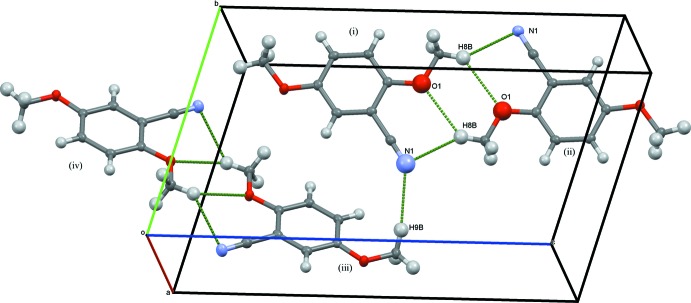
Intermolecular interactions between molecules of the title compound. [Symmetry codes: i: x,y,z; ii: 3 - x,2 - y,1 - z; iii: 2.5 - x,-1/2 + y,1/2 - z; iv: -1/2 + x,1.5 - y,1/2 + z]

**Fig. 3.**
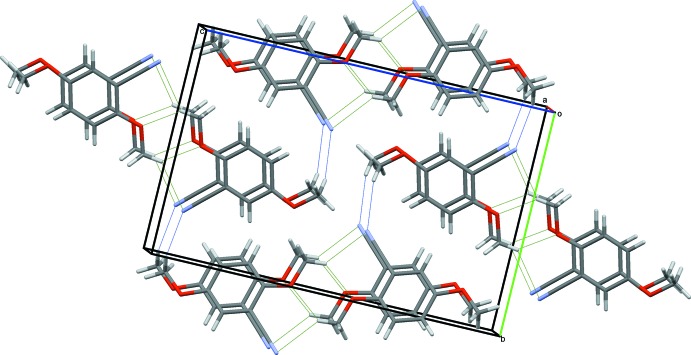
The crystal packing diagram showing the C—H···O and C—H···N intermolecular interactions between molecules within pairs (green colored) and between molecules in different strands (blue colored).

## supplementary crystallographic information

### 1. Comment

The aromatic ring (C1—C6) of the title compound is almost coplanar with non-H atoms of all substituents, with torsion angles of 3.2 (2)°, 2.5 (2)° and 178.7 (1)° for the methoxy group (C3—C2—O1—C8), for the methoxy group (C4—C5—O2—C9) and for the nitrile group (C5—C6—C1—C7), respectively. With the length of 1.1492 (19) Å, the triple bond of the nitrile group (C≡N) is at the higher end of the acceptable range of cyano bond lengths (Buschmann *et al.*, 1995; Zanotti *et al.*, 1980), but longer than in comparable alkoxy-substituted benzonitriles (Zabinski *et al.*, 2007). The molecules of the title compound arrange themselves in pairs through C8–H8B···O1 and C8–H8B···N1 interactions (Table 1, Fig. 2). In one pair, the average plane of the aromatic ring (C1—C6) of one molecule has an off-set of 0.644 (2) Å to the respective plane in the other molecule. The stacked centrosymmetric dimers form strands propagating along the *a* axis. Each pair in one strand forms four close contacts C9—H9B···N1 (Table 1) with four pairs of the four neighboring strands (Figure 3). The average plane of the aromatic ring (C1—C6) of a molecule in one strand forms an angle of 29.08 (2)° with the respective average plane of another molecule in the neighboring strand, with the molecules linked by C9—H9B···N1 close contact.

### 2. Experimental

To triphenylphosphine (870 mg, 3.3 mmol) in dry CH_2_Cl_2_ (10 ml) was added bromotrichloromethane (650 mg, 3.3 mmol), and the resulting mixture was stirred at rt for 20 min, during which the solution turned from yellow to red-brownish in color. Thereafter, 2,5-dimethoxybenzaldoxime (552 mg, 3.05 mmol) was added. The reaction mixture was kept under reflux for 25 min. Then, triphenylphosphine (870 mg, 3.3 mmol) was added, and the mixture stirred for 8 h at reflux. The cooled reaction mixture was concentrated *in vacuo* and subjected directly to column chromatography on silica gel (CH_2_Cl_2_ – hexane 5: 1) to give the title compound (195 mg, 39%) as colorless needles; m.p. 360 - 361 K (Lit. 354 - 358 K; Ushijima *et al.*, 2012); *n*_max_ (KBr/cm^-1^) 2224 (CN), 1582, 1508, 1420, 1287, 1237, 1120, 1039, 879, 815, 753, 704, 488; *d*_H_ (400 MHz, CDCl_3_) 3.77 (3*H*, s, OCH_3_), 3.87 (3*H*, s, OCH_3_), 6.89 (1*H*, d, ^3^*J* = 8.8 Hz), 7.04 (1*H*, d, ^4^*J* = 2.8 Hz), 7.07 (1*H*, dd, ^3^*J* = 8.8 Hz, ^4^*J* = 2.8 Hz); *d*_C_ (100.5 MHz, CDCl_3_) 55.9 (OCH_3_), 56.4 (OCH_3_), 101.7 (C_quat_), 112.6 (CH), 116.4 (C_quat_), 117.5 (CH), 120.8 (CH), 153.1 (C_quat_), 153.7 (C_quat_); MS (EI, 70 eV) *m/z* (%) 163 (*M*^+^, 100).

### 3. Refinement

All carbon-bound hydrogen atoms were placed in calculated positions with C—H distances of 0.95 - 0.98 Å and refined as riding with *U*_iso_(H) =xUeq(C), where *x* = 1.5 for methyl and *x* = 1.2 for all other H-atoms.

### Crystal data

**Table:**

C_9_H_9_NO_2_	*D*_x_ = 1.373 Mg m^−^^3^
*M**_r_* = 163.17	Melting point = 360–361 K
Monoclinic, *P*2_1_/*n*	Mo *K*α radiation, λ = 0.7107 Å
*a* = 3.91001 (15) Å	Cell parameters from 1290 reflections
*b* = 11.3347 (4) Å	θ = 3.6–32.0°
*c* = 17.8432 (6) Å	µ = 0.10 mm^−^^1^
β = 93.400 (3)°	*T* = 100 K
*V* = 789.40 (5) Å^3^	Block, colourless
*Z* = 4	0.60 × 0.25 × 0.23 mm
*F*(000) = 344	

### Data collection

**Table:**

Agilent SuperNova (Dual, Cu at zero, Atlas) diffractometer	1785 independent reflections
Radiation source: SuperNova (Mo) X-ray Source	1374 reflections with *I* > 2σ(*I*)
Mirror monochromator	*R*_int_ = 0.026
Detector resolution: 10.4127 pixels mm^-1^	θ_max_ = 27.5°, θ_min_ = 3.6°
ω scans	*h* = −5→4
Absorption correction: multi-scan (*CrysAlis PRO*; Agilent, 2013)	*k* = −9→14
*T*_min_ = 0.899, *T*_max_ = 1.000	*l* = −21→23
3225 measured reflections	

### Refinement

**Table:**

Refinement on *F*^2^	Primary atom site location: structure-invariant direct methods
Least-squares matrix: full	Hydrogen site location: inferred from neighbouring sites
*R*[*F*^2^ > 2σ(*F*^2^)] = 0.044	H-atom parameters constrained
*wR*(*F*^2^) = 0.116	*w* = 1/[σ^2^(*F*_o_^2^) + (0.048*P*)^2^ + 0.110*P*] where *P* = (*F*_o_^2^ + 2*F*_c_^2^)/3
*S* = 1.06	(Δ/σ)_max_ < 0.001
1785 reflections	Δρ_max_ = 0.21 e Å^−^^3^
111 parameters	Δρ_min_ = −0.24 e Å^−^^3^
0 restraints	

### Special details

**Table:**

Geometry. All e.s.d.'s (except the e.s.d. in the dihedral angle between two l.s. planes) are estimated using the full covariance matrix. The cell e.s.d.'s are taken into account individually in the estimation of e.s.d.'s in distances, angles and torsion angles; correlations between e.s.d.'s in cell parameters are only used when they are defined by crystal symmetry. An approximate (isotropic) treatment of cell e.s.d.'s is used for estimating e.s.d.'s involving l.s. planes.

### Fractional atomic coordinates and isotropic or equivalent isotropic displacement parameters (Å^2^)

**Table:**

	*x*	*y*	*z*	*U*_iso_*/*U*_eq_	
C1	1.1608 (4)	0.84580 (13)	0.32618 (8)	0.0170 (3)	
C2	1.1137 (4)	0.96337 (13)	0.34798 (7)	0.0170 (3)	
C3	0.9434 (4)	1.03957 (13)	0.29791 (8)	0.0178 (3)	
C4	0.8238 (4)	1.00071 (13)	0.22693 (8)	0.0180 (3)	
C5	0.8723 (4)	0.88435 (13)	0.20555 (8)	0.0166 (3)	
C6	1.0425 (4)	0.80699 (13)	0.25538 (7)	0.0178 (3)	
C7	1.3310 (4)	0.76401 (14)	0.37753 (8)	0.0193 (3)	
C8	1.1889 (4)	1.11088 (14)	0.44323 (8)	0.0214 (4)	
C9	0.5943 (4)	0.91476 (14)	0.08362 (8)	0.0227 (4)	
H3	0.9077	1.1192	0.3120	0.021*	
H4	0.7086	1.0541	0.1930	0.022*	
H6	1.0782	0.7274	0.2410	0.021*	
H8A	1.2963	1.1668	0.4099	0.032*	
H8B	1.2895	1.1202	0.4945	0.032*	
H8C	0.9421	1.1264	0.4425	0.032*	
H9A	0.5317	0.8708	0.0375	0.034*	
H9B	0.7498	0.9793	0.0723	0.034*	
H9C	0.3872	0.9472	0.1041	0.034*	
N1	1.4646 (4)	0.69750 (12)	0.41812 (7)	0.0264 (3)	
O1	1.2456 (3)	0.99244 (9)	0.41788 (5)	0.0202 (3)	
O2	0.7618 (3)	0.83694 (9)	0.13775 (5)	0.0219 (3)	

### Atomic displacement parameters (Å^2^)

**Table:**

	*U*^11^	*U*^22^	*U*^33^	*U*^12^	*U*^13^	*U*^23^
C1	0.0161 (7)	0.0162 (8)	0.0189 (7)	−0.0002 (6)	0.0038 (5)	0.0027 (6)
C2	0.0161 (8)	0.0185 (8)	0.0165 (7)	−0.0017 (6)	0.0025 (5)	0.0012 (6)
C3	0.0184 (8)	0.0147 (7)	0.0204 (7)	0.0000 (6)	0.0026 (6)	−0.0003 (6)
C4	0.0178 (8)	0.0170 (7)	0.0194 (7)	0.0008 (6)	0.0024 (6)	0.0039 (6)
C5	0.0157 (7)	0.0176 (8)	0.0165 (7)	−0.0028 (6)	0.0020 (5)	0.0008 (6)
C6	0.0169 (8)	0.0164 (7)	0.0206 (7)	−0.0007 (6)	0.0041 (6)	−0.0001 (6)
C7	0.0210 (8)	0.0174 (8)	0.0196 (7)	−0.0005 (7)	0.0030 (6)	−0.0018 (6)
C8	0.0260 (9)	0.0184 (8)	0.0196 (7)	0.0012 (7)	−0.0007 (6)	−0.0027 (6)
C9	0.0256 (9)	0.0229 (8)	0.0189 (7)	−0.0011 (7)	−0.0029 (6)	0.0031 (6)
N1	0.0334 (9)	0.0212 (7)	0.0243 (7)	0.0046 (6)	−0.0004 (6)	0.0003 (6)
O1	0.0258 (6)	0.0172 (6)	0.0171 (5)	0.0027 (5)	−0.0029 (4)	−0.0009 (4)
O2	0.0281 (6)	0.0196 (6)	0.0174 (5)	0.0000 (5)	−0.0033 (4)	0.0002 (4)

### Geometric parameters (Å, º)

**Table:**

C2—C1	1.404 (2)	C8—H8C	0.9800
C2—C3	1.384 (2)	C8—H8B	0.9800
C3—C4	1.395 (2)	C8—H8A	0.9800
C3—H3	0.9500	C9—H9C	0.9800
C4—H4	0.9500	C9—H9B	0.9800
C5—C4	1.389 (2)	C9—H9A	0.9800
C5—C6	1.390 (2)	O1—C8	1.4381 (18)
C6—C1	1.391 (2)	O1—C2	1.3616 (17)
C6—H6	0.9500	O2—C9	1.4370 (18)
C7—N1	1.1492 (19)	O2—C5	1.3700 (17)
C7—C1	1.439 (2)		
			
C1—C6—H6	119.9	H8A—C8—H8B	109.5
C2—C1—C7	119.92 (13)	H8B—C8—H8C	109.5
C2—C3—C4	120.75 (14)	H9A—C9—H9C	109.5
C2—C3—H3	119.6	H9A—C9—H9B	109.5
C2—O1—C8	117.18 (11)	H9B—C9—H9C	109.5
C3—C4—H4	119.8	N1—C7—C1	179.12 (16)
C3—C2—C1	118.65 (13)	O1—C8—H8C	109.5
C4—C3—H3	119.6	O1—C8—H8B	109.5
C4—C5—C6	119.42 (13)	O1—C8—H8A	109.5
C5—C4—H4	119.8	O1—C2—C1	115.77 (13)
C5—C4—C3	120.35 (14)	O1—C2—C3	125.57 (14)
C5—C6—C1	120.16 (14)	O2—C9—H9C	109.5
C5—C6—H6	119.9	O2—C9—H9B	109.5
C5—O2—C9	117.39 (11)	O2—C9—H9A	109.5
C6—C1—C7	119.42 (13)	O2—C5—C4	125.03 (13)
C6—C1—C2	120.67 (14)	O2—C5—C6	115.55 (13)
H8A—C8—H8C	109.5		

### Hydrogen-bond geometry (Å, º)

**Table:**

*D*—H···*A*	*D*—H	H···*A*	*D*···*A*	*D*—H···*A*
C8—H8*B*···O1^i^	0.98	2.65	3.428 (2)	136
C8—H8*B*···N1^i^	0.98	2.73	3.504 (2)	136
C9—H9*B*···N1^ii^	0.98	2.71	3.640 (2)	158

Symmetry codes: (i) −*x*+3, −*y*+2, −*z*+1; (ii) −*x*+5/2, *y*+1/2, −*z*+1/2.
